# Seasonal population dynamics of the primary yellow fever vector *Haemagogus leucocelaenus* (Dyar & Shannon) (Diptera: Culicidae) is mainly influenced by temperature in the Atlantic Forest, southeast Brazil

**DOI:** 10.1590/0074-02760200218

**Published:** 2020-07-20

**Authors:** Dinair Couto-Lima, Cecilia S Andreazzi, Paulo José Leite, Maria Ignez Lima Bersot, Jeronimo Alencar, Ricardo Lourenço-de-Oliveira

**Affiliations:** 1 Fundação Oswaldo Cruz-Fiocruz, Instituto Oswaldo Cruz, Laboratório de Mosquitos Transmissores de Hematozoários, Rio de Janeiro, RJ, Brasil; 2 Fundação Oswaldo Cruz-Fiocruz, Instituto Oswaldo Cruz, Laboratório de Biologia e Parasitologia de Mamíferos Silvestres Reservatórios, Rio de Janeiro, RJ, Brasil; 3 Secretaria Municipal de Saúde de Nova Iguaçu, RJ, Brasil; 4 Fundação Oswaldo Cruz-Fiocruz, Instituto Oswaldo Cruz, Laboratório de Diptera, Rio de Janeiro, RJ, Brasil

**Keywords:** mosquito ecology, oviposition, rainfall, temperature, yellow fever, Rio de Janeiro

## Abstract

**BACKGROUND:**

Southeast Brazil has recently experienced a Yellow Fever virus (YFV) outbreak where the mosquito *Haemagogus leucocelaenus* was a primary vector. Climatic factors influence the abundance of mosquito vectors and arbovirus transmission.

**OBJECTIVES:**

We aimed at describing the population dynamics of *Hg. leucocelaenus* in a county touched by the recent YFV outbreak.

**METHODS:**

Fortnightly egg collections with ovitraps were performed from November 2012 to February 2017 in a forest in Nova Iguaçu, Rio de Janeiro, Brazil. The effects of mean temperature and rainfall on the *Hg. leucocelaenus* population dynamics were explored.

**FINDINGS:**

*Hg. leucocelaenus* eggs were continuously collected throughout the study, with a peak in the warmer months (December-March). The climatic variables had a time-lagged effect and four weeks before sampling was the best predictor for the positivity of ovitraps and total number of eggs collected. The probability of finding > 50% positive ovitraps increased when the mean temperature was above 24ºC. The number of *Hg. leucocelaenus* eggs expressively increase when the mean temperature and accumulated precipitation surpassed 27ºC and 100 mm, respectively, although the effect of rainfall was less pronounced.

**MAIN CONCLUSIONS:**

Monitoring population dynamics of *Hg. leucocelaenus* and climatic factors in YFV risk areas, especially mean temperature, may assist in developing climate-based surveillance procedures to timely strengthening prophylaxis and control.

From 2016-2019, yellow fever has caused an unprecedented outbreak in Brazil.^1^ It was consequence of a southward epizootic spreading wave of Yellow Fever virus. (YFV) started in the Amazon in 2014.^2^ ^,^ ^3^ Almost 98% of the recorded 2,259 human cases and 773 deaths occurred in the Southeast region, in sites under influence of the Cerrado and Atlantic Forest biomes.^4^ The urban *Aedes aegypti* (Linnaeus)*-*borne transmission has been eradicated for more than 70 years from the country.^5^ ^,^ ^6^ Thus, human infections in this outbreak were typically acquired in the zoonotic sylvatic transmission cycle where non-human primates (NHPs) are the amplification vertebrate hosts and *Haemagogus* and *Sabethes* mosquitoes the primary and secondary vectors, respectively.^1^ ^,^ ^7^ ^,^ ^8^

*Haemagogus leucocelaenus* (Dyar & Shannon) (Diptera: Culicidae: Aedini) is a sylvatic primatophilic mosquito distributed from Panama to northern Argentina and Uruguay, whose immature stages develops essentially in water contained in tree-holes.^9^ ^,^ ^10^ The eggs of *Hg. leucocelaenus* are laid on moist vegetal substrates near water level, where they resist to desiccation and keep the embryos viable outside the water for months^9^ ^,^ ^11^ Females of *Hg. leucocelaenus* are active during daytime, when they may attack NHPs at the tree canopies and humans on the forest ground or modified open fields.^12^ ^,^ ^13^ ^,^ ^14^ The species has been suggested as YFV vector in previous transmission emergences in Brazil and Colombia.^12^ ^,^ ^15^ ^,^ ^16^ ^,^ ^17^ ^,^ ^18^ ^,^ ^19^ ^,^ ^20^

Entomological surveys conducted during the 2016-2019 outbreak in the southeast Brazil incriminated *Hg. leucocelaenus* as YFV primary vector together with *Hg. janthinomys* Dyar, while some *Aedes* and *Sabethes* species were considered to play a secondary or local role in transmission.^14^ ^,^ ^21^ It was noticed that *Hg. leucocelaenus* was widespread and abundant in the YFV foci, with detections of high rates of natural infections.^14^ ^,^ ^21^ In laboratory, a population of *Hg. leucocelaenus* from southeast Brazil has proven to be competent to transmit YFV belonging to distinct lineages of the South America I as well as the West-Africa genotypes.^22^ Besides its role in YFV transmission, the abovementioned *Hg. leucocelaenus* population was experimentally competent to transmit Chikungunya virus,^23^ and one amplicon of putative DENV-1 was found in one pool of this species collected in northeast Brazil.^24^

Climatic factors influence the abundance and activity of mosquito vectors, which in turn affect arbovirus transmission such as YFV.^25^ ^,^ ^26^ ^,^ ^27^ Here, we evaluated the influence of climatic variables such as temperature and rainfall in the seasonal dynamics of *Hg. leucocelaenus* during a long-term egg collection conducted in an Atlantic Forest area in a county of southeast Brazil touched by the recent YFV outbreak.

## MATERIALS AND METHODS

*Study area* - The study was conducted in Parque Natural Municipal de Nova Iguaçu (PNMNI) (22º46’45”S 43º27’23”W), a conservation area of 1,100 hectares of the Atlantic Forest biome at the northwest flank of the Gericinó massif, adjacent to the periurban zone of the municipality of Nova Iguaçu, at 35 km from the city of Rio de Janeiro, Brazil.^28^ As other counties in southeast Brazil, the recent YFV outbreak reached Nova Iguaçu in 2017-2018, with records of autochthonous human cases, numerous epizooties of NHPs and natural infections in sylvatic mosquitoes.^14^ ^,^ ^29^ The forest in PNMNI cover an essentially mountain area, with altitude varying from 150-956 m. The local climate is classified as Aw (Köppen-Geiger classification) with rainy summer (December to March) and dry winter (June-September); the average temperature and annual rainfall are 23.4ºC and 1408 mm, respectively.^30^

*Mosquito collection* - Mosquito collections were approved by local environmental authorities (PNMNI license 001/14-15; SISBIO-MMA licenses 37362-2 and 012/2016). A total of 20 ovitraps^31^ containing ~ 300 mL of water from a local source and leaf litter, and three plywood paddles (Eucatex®, Brazil) as oviposition support was suspended on tree branches at a height of 3-12 m. Ovitraps were distributed in the forest at different distances from each other (3.3-22.9 m) and from the edge of a narrow path (0-173.2 m) that runs roughly parallel to a non-perennial stream. The number of used ovitraps per sampling and their locations did not change during the entire study. Paddles were sampled from November 2012 to February 2017. At each fortnightly sampling, the used paddles were changed by new ones, and brought to the laboratory, allowed to dry slowly for ten days in an insectary (26 ± 1ºC; 70 ± 10% RH) and examined to egg counting. Eggs were then hatched by immersing the paddles twice in dechlorinated tap water for two consecutive days. Larvae were reared in pans (~ 50 larvae/pan measuring 25 x 25 x 10 cm) containing 1 L of dechlorinated tap water, supplemented with yeast powder and shed leaves, renewed every 2-3 days. Emerged adults were transferred to cubic (30 cm) mesh cages supplied with 10% honey solution in the mentioned insectary, and soon morphologically identified to species according to Consoli and Lourenço-de-Oliveira.^25^

*Climate data* - To evaluate the influence of temperature and rainfall in the seasonal dynamics of *Hg. leucocelaenus* oviposition in the ovitraps we used data obtained by the closest meteorological stations of Instituto Nacional de Meteorologia (INMET) with availability of the required climate data for the study period. Accordingly, daily mean temperatures were available from the Rio de Janeiro station (A636; OMM: 83743, 18 km from PNMNI), while regarding daily rainfall we used the average of the records of the two nearest stations from the collecting site: the Duque de Caxias - Xerém (A603; OMM: 86877, 28 km from PNMNI) and Ecologia Agrícola - Seropédica (A601; OMM: 86878; 42 km) stations.

*Data analysis* - We calculated two indices: (a) the proportion of positive ovitraps per sampling (“positive” means at least one egg was found on paddles) and (b) the total number of eggs collected in all ovitrap per fortnightly sampling. Exploratory data analysis and model validation was conducted following Zuur et al.^32^

We explored the effects of mean temperature and rainfall (fixed effects) on the proportion of positive ovitraps using generalised linear mixed models (GLMMs)^32^ with a binomial error structure and logit link. We considered as fixed predictor variables the mean temperature and total rainfall accumulated in 1-6 weeks prior to the sampling event as well as the height of the ovitrap and its distance to the edge of the forest. The ovitrap was considered as a random factor to control for variation among ovitraps. Such variation could be related to the height of the ovitrap and its distance from the edge of the forest, so we specified them as random slopes of the random effect.

Since the variance of the total number of eggs (2932) was much greater than its mean (31), characterising over-dispersion, the Negative Binomial probability distribution was chosen instead of the Poisson distribution, the typical probability distribution for modeling counting data. The effects of mean temperature and rainfall (fixed effects) on the total number of eggs were analysed using GLMMs^32^ with a negative binomial error structure and logarithm link. In the same way as for the proportion of positive ovitrap models (see above), we considered as fixed predictor variables the mean temperature and total rainfall accumulated in 1-6 weeks prior to the sampling event and the height of the ovitrap and its distance to the edge of the forest. The ovitrap was also considered as a random factor.

In all cases, we started by building a full GLMM, then created nested GLMMs by evaluating the significance of predictors with Wald chi-square tests and dropping the non-significant individual predictors (p > 0.05) based on differences in model fit.^32^ We ensured the predictors were not correlated with each other.^33^ We ranked all candidate models by the lowest Akaike information criterion (AIC) and evaluated their relative likelihoods using AIC weights,^34^ considering a null model with only the intercept as a benchmark. The most parsimonious model was selected as the one with the lowest AIC. Models with the difference in AIC < 2 were considered equally plausible. We also calculated the area under the receiver operating characteristic (ROC) curves - the area under the ROC curve (AUC)^35^ - to find the combination of predictor variables that maximises the probability of finding positive ovitraps. Finally, we followed the protocol to validate the most parsimonious GLMMs by inspecting Q-Q plots and plots of residuals against fitted data and deviance residuals against predicted data.^36^ The GLMMs were carried out with the “glmer.nb” and “glmer” functions in “lme4” package in software R version 3.6.2.

To investigate potential nonlinearities on the effects of rainfall and temperature on total number of eggs, we also fitted a set of generalised additive mixed models (GAMMs) with log link function and negative binomial distribution (for the total number of eggs) and binomial distribution (for the positivity of the ovitrap). GAMMs are an extension of GLMMs that allows for the inclusion of nonparametric smoothing terms in the place of the constant parameters. By plotting the fitted smooth terms versus the predictor, one may investigate the nature of the relationship between the predictor and the outcome variable, detecting potential nonlinearities.

## RESULTS

*Haemagogus leucocelaenus* was the only species of *Haemagogus* detected in the area and by far the predominant mosquito ovipositing in the settled ovitraps throughout the collection period. The other two species occasionally found [*Aedes albopictus* (Skuse) and *Aedes terrens* (Walker)] were not considered.

**Fig. 1:**
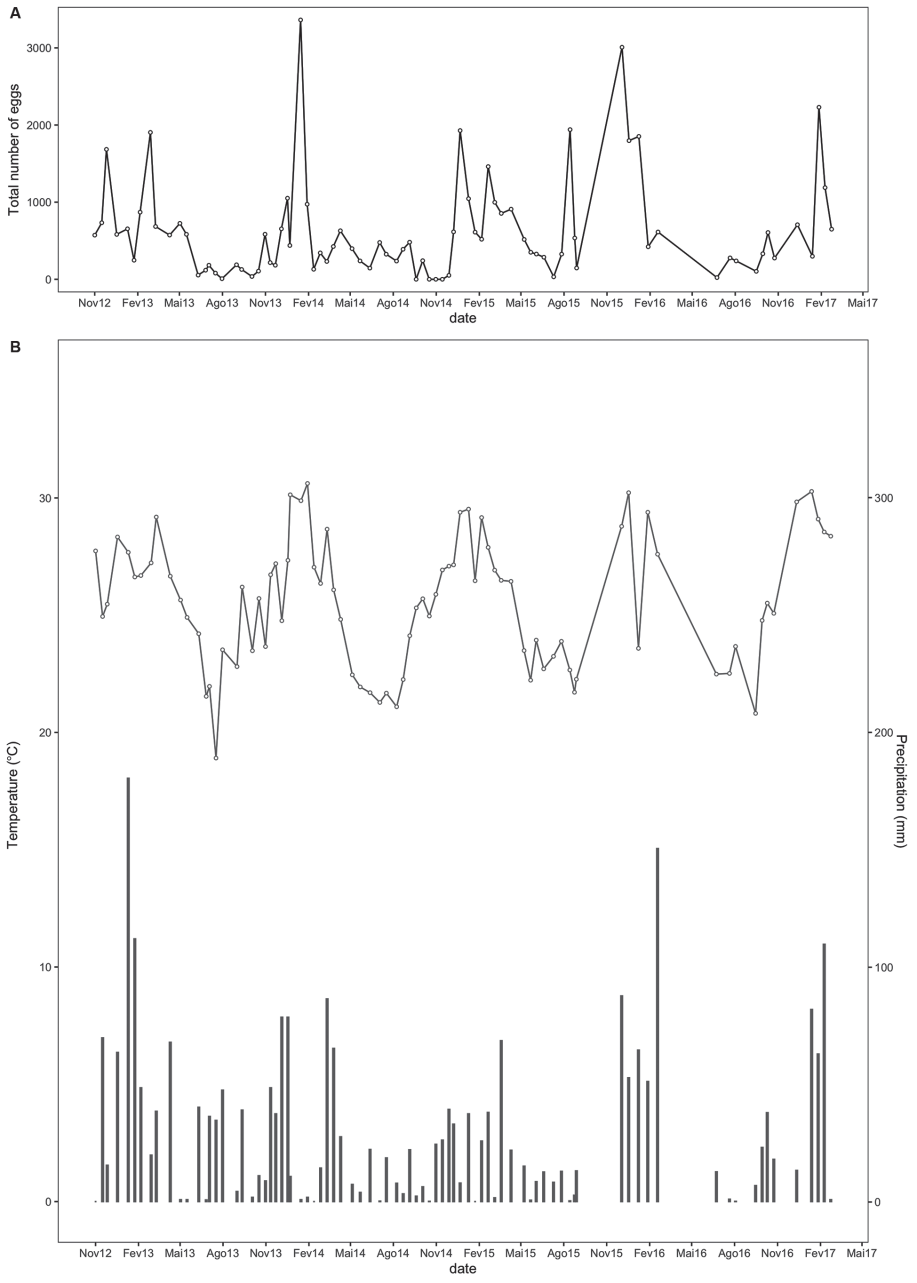
time series with (A) Sum of the total number of eggs collected in the 20 trap stations on each sampling event. (B) Time series of the mean temperature and accumulated rainfall considering the period in-between the sampling events.

**Fig. 2:**
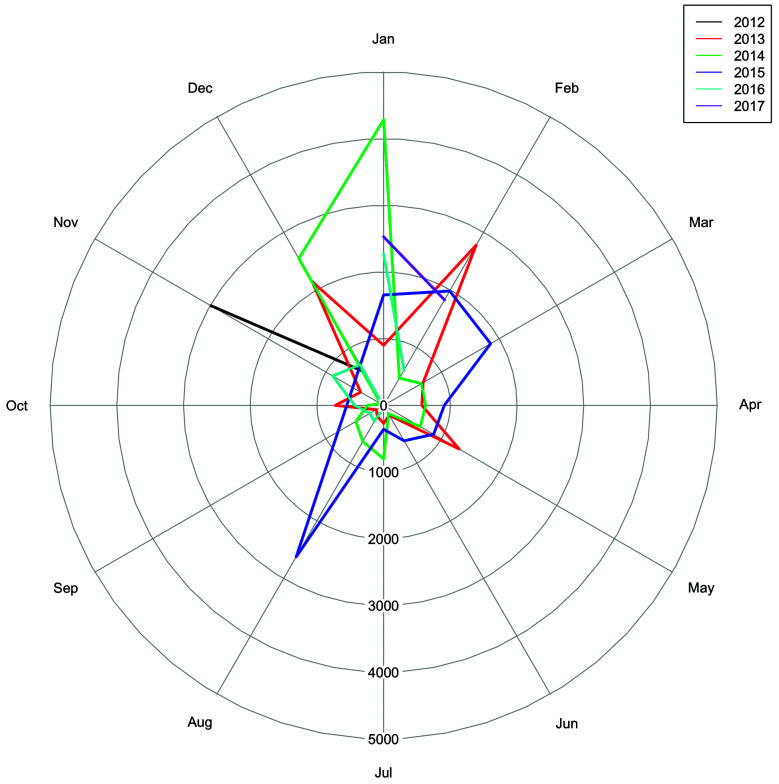
circular histogram for the sum of the total number of eggs collected per month during the studied period. Each colour represents the sampled year.

**Fig. 3:**
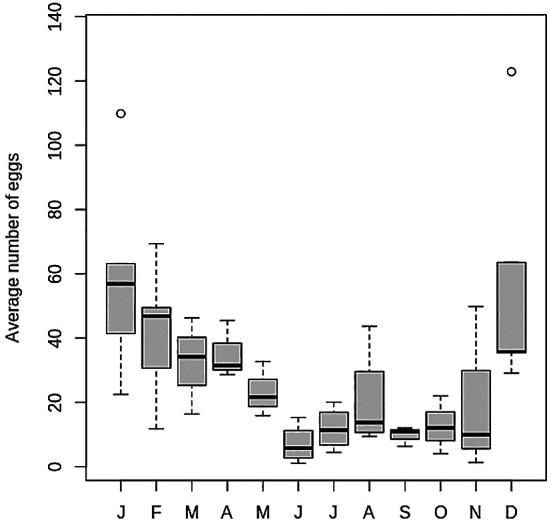
distribution of the average number of eggs collected in the 20 ovitraps for each month considering the entire study (2012-2017). Thick lines within boxes represent median values for the average number of eggs collected per month in each year. Upper and lower limits of boxes represent 1st and 3rd quartiles, respectively.

Oviposition of *Hg. leucocelaenus* was recorded in every sampling throughout the years (Fig. 1), gathering a total of 50,921 eggs the entire study. Higher egg amounts were usually recorded in the warmer months (December-March) than in those with lower mean temperatures (June-October) (Fig. 2), which respectively coincide with the periods of higher and lower rainfalls (Fig. 1). The exception was a peak reported in August 2015 (Figs 1, 2). Regardless of the sampling year, the mean number of eggs collected from April to October was consistently low, although the data for August differed from the pattern influenced by the apparently atypical collections in 2015 (Fig. 2). This general distribution tendency is confirmed when we analysed the monthly pattern of average number of eggs gathered in each month from 2012 to 2017 (Fig. 3). Again, the number of collected eggs was higher in January and December than in the rest of the year (particularly in June). Greater amplitude in egg counting was observed in the months of transition between summer and autumn (March) and between spring and summer (November).

**Fig. 4:**
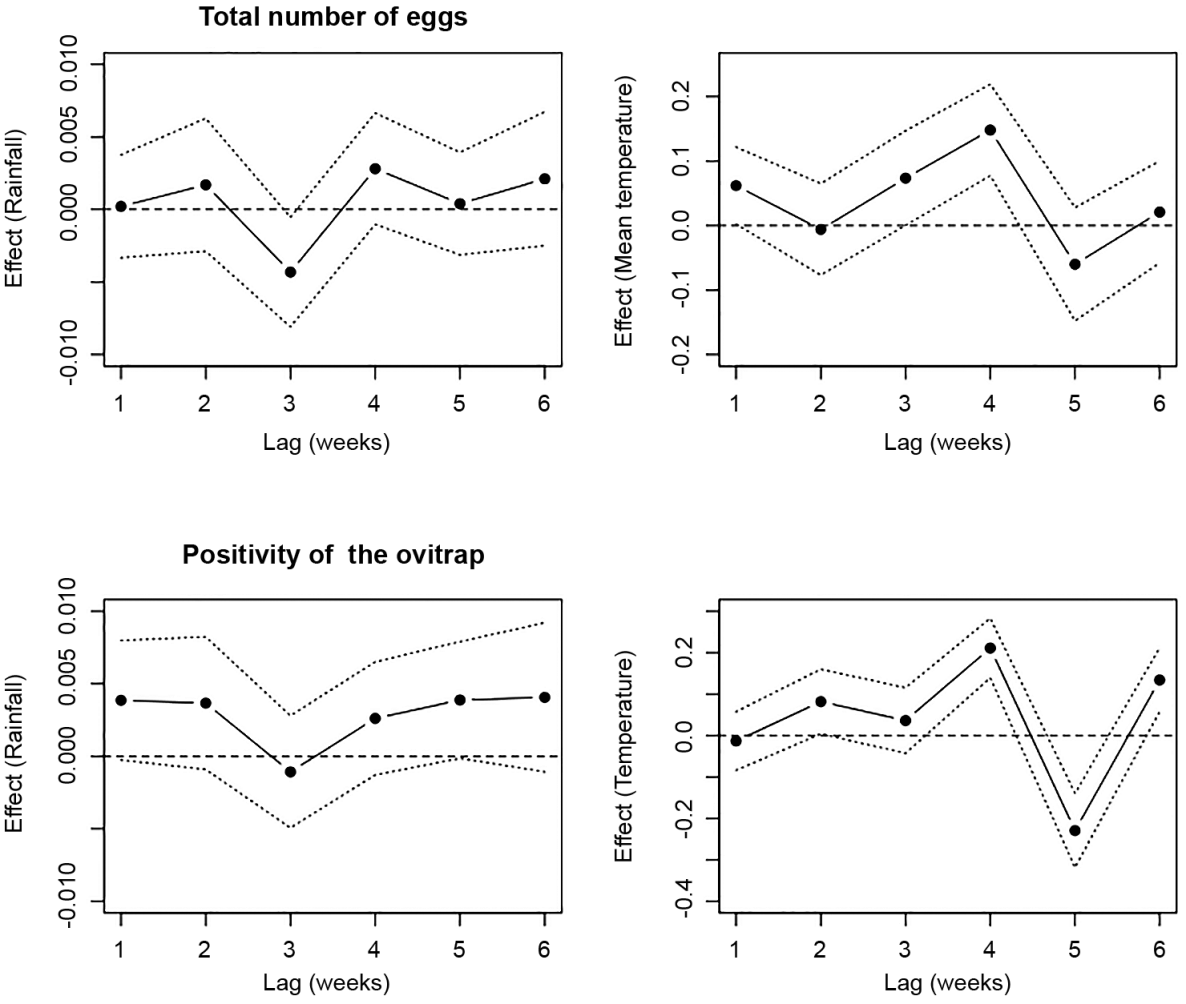
lag distributed effect of temperature and rainfall on considering the full models the total number of eggs produced (top) and the positivity of the ovitraps (bottom). Dotted lines indicated 95% confidence interval.

We evaluated the influence of accumulated rainfall and mean temperature recorded from one to six weeks before sampling events in both GLMMs and GAMMs models. The accumulated rainfall of three weeks before a sampling event had a significant negative effect on the total number of collected eggs (Fig. 4). No significant positive influence of rainfall accumulated during any time lag on the amount of laid egg in the ensemble of ovitraps was found (Fig. 4). In contrast, the mean temperature recorded during the three and four weeks before a sampling event had a significant and positive effect on the total number gathered eggs (Fig. 4). When considering the probability of finding a positive ovitraps, it was noticed that the accumulated rainfall considering 1-6-weeks’ time lags did not affected positivity (Fig. 4). On the other hand, the mean temperature of four and six weeks before the sampling event had a significant and positive effect on the positivity of the ovitraps, while a negative effect was found when considering a five-week time lag (Fig. 4).

**Fig. 5:**
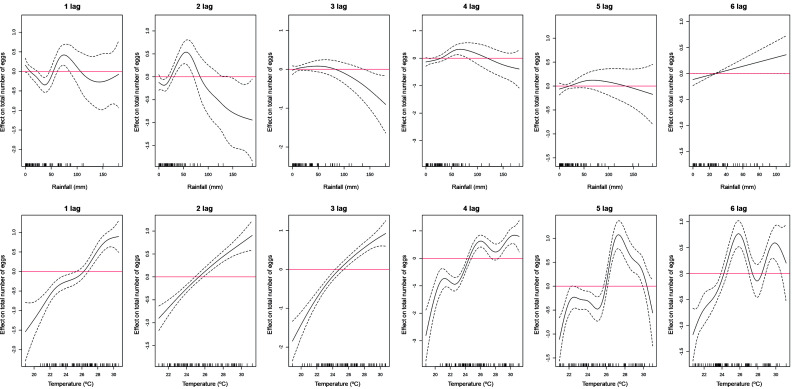
smooth effect of temperature and rainfall at lag 1-6 weeks on the total number of collected eggs. Dotted lines indicated 95% confidence interval.

**Fig. 6:**
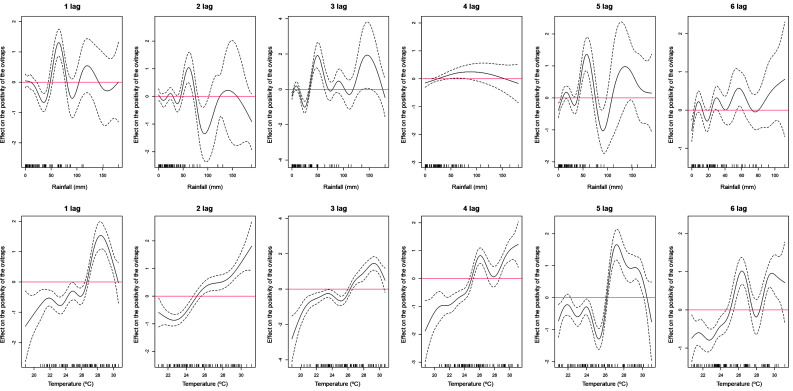
smooth effect of temperature and rainfall at lag 1-6 weeks on the positivity of the ovitraps. Dotted lines indicated 95% confidence interval.

Rainfall had a nonlinear effect either on the number of collected eggs or the positivity of ovitraps at 1-6 weeks’ time lags of samplings (Figs 5, 6). In contrast, temperature had a linear or relatively linear effect on the number of laid eggs in the ovitraps at 1-4 weeks, but became nonlinear after at 5-6 weeks’ time lag (Fig. 5). Considering positivity of ovitraps, temperature had a nearly linear effect only in the interval of 2-4 weeks’ time lag of a samplings (Fig. 6).

All the five most plausible models explaining the total number of eggs gathered considered a time lag of four weeks and included mean temperature, rainfall and the height of the ovitrap (Table I). The distance from the forest edge was not included in any of the plausible models. For the positivity of ovitraps we also found that temperature and rainfall considering a time lag of four weeks were the explanatory variables included in all the most plausible models (Table III).

**Fig. 7:**
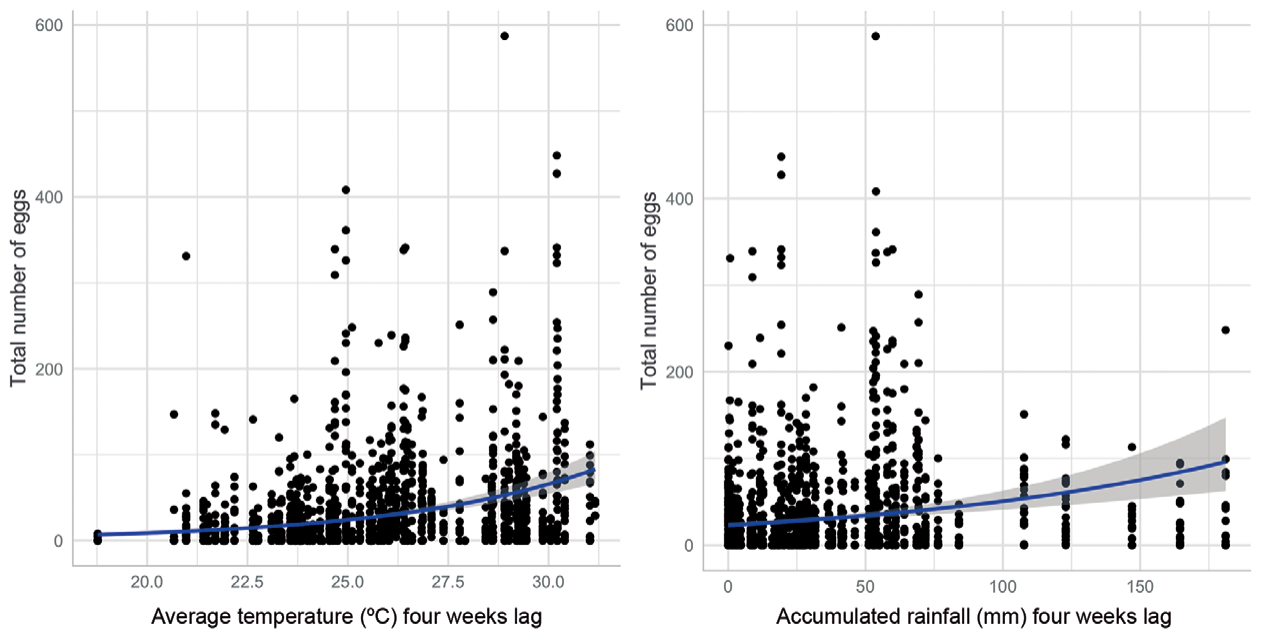
effect of temperature and rainfall (four weeks lag) on the total number of eggs collected considering the 20 ovitraps. The line represents the predicted number of eggs.

**Fig. 8:**
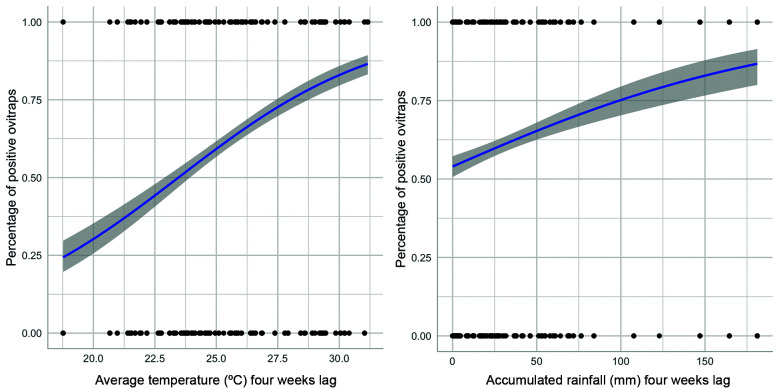
effect of temperature and rainfall (four weeks lag) on the positivity of the ovitraps. The line represents the predicted percentage of positive ovitraps.

The estimates of the most plausible models for total number of eggs and positivity of ovitraps are in Tables II and IV, respectively. When evaluating the effect of temperature and rainfall with four weeks the model predicted a greater increase in the number of eggs when the mean temperature was above 27ºC and when the accumulated rainfall was above 100 mm (Fig. 7). However, the effect of rainfall was less pronounced than the effect of mean temperatures. In the same direction, we found that the probability of finding more than 50% of ovitraps containing eggs was higher when the mean temperatures during the 4-weeks’ time lag before sampling was above 24ºC (Fig. 8). This model also showed that we would expect to find more than 75% of the ovitraps with at least one egg when the accumulated rainfall during the 4-weeks’ time lag before sampling was above 100 mm (Fig. 8). The height of the ovitrap was included in the second most plausible model for the total number of eggs, but with a non-significant effect (β = -0.06, p = 0.36).

**TABLE I:** Comparison of the top five candidate generalised linear mixed models (GLMMs) for the total number of eggs

Model	Lag	AIC	ΔAIC	k	wAIC
rainfall + temperature + (1 | ovitrap)	4	12230.34	0	5	0.54
rainfall + temperature + height + (height | ovitrap)	4	12231.2	0.87	8	0.35
rainfall + temperature + distance + (distance | ovitrap)	4	12235.1	4.76	8	0.05
temperature + (1 | ovitrap)	4	12236.34	5.99	4	0.03
temperature + height + (height | ovitrap)	4	12270.3	6.8	7	0.02
1 + (1 | ovitrap)	null	12354.9	124.6	3	< 0.001

Lag: the time lag og the climatic variable (in weeks); AIC: Akaike information criterion; k: number of model parameters; ΔAIC: difference between the AIC of a given model and that of the best model; wAIC: Akaike weights. Marked in gray are the most plausible models (ΔAIC < 2).

**TABLE II:** Estimated parameters of the most parsimonious generalised linear mixed models (GLMMs) (Table I) describing the effects of temperature and rainfall with four weeks’ time lag on the total number of eggs, considering ovitrap as random variable

Intercept	Estimate	SE	z-value	p-value
	-2.23	0.53	-4.23	< 0.0005
Fixed effects				
Temperature	0.20	0.02	9.99	< 0.0005
Rainfall	0.004	0.001	2.71	0.007
Random effects	Variance	SDev		
Ovitrap	0.31	0.56		

SE: standard error; SDev: standard deviation

**TABLE III:** Comparison of the top five candidate generalised linear mixed models (GLMMs) for the positivity of the ovitraps

Model	Lag	AIC	ΔAIC	k	wAIC	AUC ROC
rainfall + temperature + (1 | ovitrap)	4	1988.3	0	4	0.79	0,7393147
rainfall + temperature + height + (height | ovitrap)	4	1992.06	3.75	7	0.12	0,7389699
rainfall + temperature + distance + (distance | ovitrap)	4	1993.36	5.06	7	0.06	0,7390462
temperature + (1 | ovitrap)	4	1995.09	6.79	3	0.02	0,73572
rainfall + temperature + height + distance + (height | ovitrap) + (distance | ovitrap)	4	1998.48	10.18	11	5	0,7389089
1 + (1 | ovitrap)	null	2161.4	173.1	2	< 0.001	0,6512692

Lag: the time lag og the climatic variable (in weeks); AIC: Akaike information criterion; k: number of model parameters; ΔAIC: difference between the AIC of a given model and that of the best model; wAIC: Akaike weights; AUC ROC: area under the ROC curve. Marked in gray are the most plausible models (ΔAIC < 2).

**TABLE IV:** Estimated parameters of the most parsimonious generalised linear mixed models (GLMMs) (Table III) describing the effects of temperature and rainfall with four weeks’ time lag on the positivity of the ovitraps, considering ovitrap as random variable

Intercept	Estimate	SE	z-value	p-value
-5.73	0.56	-10.31	< 0.0005
Fixed effects				
Temperature	0.24	0.02	10.95	< 0.0005
Rainfall	0.005	0.001	2.9	0.004
Random effects	Variance	SDev		
Ovitrap	0.33	0.57		

SE: standard error; SDev: standard deviation.

## DISCUSSION

In Brazil, humans are contaminated by YFV during epizooties, by the bite of infected sylvatic mosquitoes, primarily *Hg. leucocelaenus* and *Hg. janthinomys*.^14^ ^,^ ^17^ From the entomological point of view, only preventive measures to avoid mosquito biting when into or near epizootic forests by using repellents and personal protective equipment are plausible. These forest mosquitoes breed essentially in rather cryptic tree holes.^9^ ^,^ ^25^ Thus, YFV control strategy based on the fight against their adult and immature forms is unfeasible.

On the other hand, understanding the population dynamics of YFV primary vectors such as *Hg. leucocelaenus* and the climatic variables influencing this dynamic may help in defining expanded risk areas, predicting silent virus circulation and NHP epizooties, timely implementing adequate prophylaxis and control strategies such as intensification of local vaccination campaigns in risk areas. In the present study, we described population dynamics of *Hg. leucocelaenus* based on a long-term egg collection in a forest located in a municipality of southeastern Brazil affected by the recent YFV outbreak. In summary, our data evidenced that *Hg. leucocelaenus* has a pronounced seasonal population dynamics expressively influenced by variations of climatic factors, where temperature has a major role.

Some Aedinii species whose eggs are resistant to desiccation may disappear in adulthood stage during some months in the unfavorable season, persisting as embryos. It is not the case of *Hg. leucocelaenus* in the Atlantic Forest. Interestingly, in the present study eggs of *Hg. leucocelaenus* were detected in every sampled month from November 2012 to March 2017. *Hg. leucocelaenus* females need to take at least one blood meal to sustains its anautogenous reproduction and may retain eggs of a previous gonotrophic cycle in their ovaries when seeking for a new blood meal.^37^ So, even though fluctuating in number, the uninterrupted encounter of eggs of *Hg. leucocelaenus* in the area indirectly illustrates that this species maintains haematophagic activity throughout the months in the PNMNI forest, regardless of climate conditions.

Climate in the southeast Brazil, particularly mean temperature and rainfall are adequate for maintenance of YFV transmission throughout the year in almost anywhere in this region.^38^ Indeed, human cases and confirmed YFV epizooties in NHPs were recorded every month, except September in the 2017-2018 outbreak in southeast Brazil, although reports peaked during the rainy summer, December-March.^39^ Coincidently, regardless the year of sampling, higher numbers of eggs of *Hg. leucocelaenus* were usually recorded from December to March than in those months with lower mean temperatures and rainfall, from June to October in PNMNI, with larger amplitudes in the number of gathered eggs in March and November, months of transition between summer and autumn and spring and summer, respectively. This pattern of monthly egg collection and biting activity of *Hg. leucocelaenus* has been described in other sites of Atlantic Forest in southeast^11^ ^,^ ^40^ ^,^ ^41^ and in the Cerrado in centre-west Brazil^42^ ^,^ ^43^ as well as in Trinidad.^37^ ^,^ ^44^ Moreover, egg counting in January was significantly higher in the PNMNI forest, which coincided with the peak month of human case records within the 2017-2018 epidemic wave in the southeast areas under influence of the Atlantic Forest.^39^

Noteworthy, collections of *Hg. leucocelaenus* made simultaneously in three Atlantic Forest sites, in 2015-2016, that is, before the arrival of the YFV epizootic wave in this part of southeast Brazil, curiously revealed distinct dynamics from the aforementioned pattern. Accordingly, in PNMNI and Jacarapaguá (~ 20 km away),^45^ unexpected peaks were respectively recorded in August 2015 and October 2015, in the dry-cold season, while in Casimiro de Abreu (~ 130 km from PNMNI)^41^ the egg counting peaked in December 2015, in the rainy summer as expected. Thus, variations in the population dynamics of *Hg. leucocelaenus* can occur in the same type of biome a few kilometers apart. Coincidentally, an El Niño phenomenon categorised as very strong was recorded in 2015-2016.^46^

The combination of high average temperature and precipitation recorded during the rainy summer favors YFV transmission and geographical spread of epizootic waves by positively influencing mosquito egg hatching and accelerating larval development.^25^ ^,^ ^26^ When we evaluated the influence of accumulated rainfall and mean temperature recorded from one to six weeks before samplings, rainfall accumulated during any time lag did not influenced the amount of eggs laid by *Hg. leucocelaenus* at PNMNI*.* Moreover, but unlike Casimiro de Abreu,^41^ precipitation had a nonlinear effect either on the number of collected *Hg. leucocelaenus* eggs or the detection of eggs in ovitraps at any time lag in PNMNI. The height of the ovitrap in the tree canopies in PNMNI had a negative but very small effect on the total number of *Hg. leucocelaenus* eggs all 1-6 weeks’ time lags. It seems that this species may lay eggs and bite in a large range of heights in the Atlantic Forest.^11^ ^,^ ^40^ ^,^ ^44^ This ability to move vertically in the forest favors zoonotic transmission of pathogens from infected arboreal animals such as infected NHPs to humans.

In contrast to other tested variables, temperature showed to considerably influence in the population dynamics of *Hg. leucocelaenus*. The mean temperature recorded during four weeks before samplings had a significant and positive effect both on the total number eggs and the probability of finding a positive ovitrap. Also, temperature had a linear or relatively linear effect on the number of collected eggs and the positivity of ovitraps specially in the interval of 2-4 weeks’ time lag in PNMNI. These results are similar to observations made in another Atlantic Forest area, where mean temperature but not rainfall recorded in the same month of sampling was positively related to number of collected *Hg. leucocelaenus* eggs.^11^

It has been demonstrated that higher mean temperatures induce greater mosquito abundance and biting activity,^25^ besides reducing the duration of the extrinsic incubation period of YFV, that is the time elapsed between taking an infective bloodmeal and the delivery of viral particles in the saliva of a competent mosquito vector.^47^ ^,^ ^48^ ^,^ ^49^ An increase in temperature, but also of rainfall was noticed the month preceding the 2000 YFV outbreak in Brazil.^50^ Thus, temperature has an important influence in YFV circulation, and monitoring mean temperature in risk areas can help in predicting enhancement of YFV activity. Accordingly, when analysing the model predictions, we found that the probability of having more than 50% of ovitraps containing at least one egg of *Hg. leucocelaenus* was higher when the mean temperatures during the 4-weeks’ time lag before sampling is above 24ºC. This data may be taken as an indirect sign of the start of increased biting activity of *Hg. leucocelaenus* in the area. Moreover, we verified an expressive increase in the number of eggs of *Hg. leucocelaenus* four weeks after the mean temperature surpasses 27ºC. Mean weekly temperatures above 22-24ºC was found to be strongly associated with high *Ae. aegypti* abundance and consequently with an increased risk of dengue transmission in Rio de Janeiro.^51^

Although the effect of rainfall is less pronounced than that of mean temperatures in *Hg. leucocelaenus* population dynamics, we found that more than 75% of the ovitraps became positive and the number of eggs increased when the accumulated rainfall in four weeks’ time lag was above 100 mm. Very distinctly from the eggs of *Hg. janthinomys*, those of *Hg. leucocelaenus* hatch mostly after the first immersion in water.^52^ Hence, we assume that rainfall above 100 mm would raise the volume of water in the tree holes sufficiently to cover and lead to the immediate hatching of most of the existing viable eggs of *Hg. leucocelaenus*. We suppose that with the simultaneous increase in mean temperatures (> 27ºC), the generation of adults that emerged in this batch would rapidly develop and increase the number of accumulated eggs detected four weeks later.

In conclusion, monitoring population dynamics of *Hg. leucocelaenus* in risk areas and expanded risk areas is an important component in the YFV surveillance system. Moreover, our data suggest that, besides monitoring mean temperature, and secondarily rainfall may assist in constructing climate-based surveillance procedures to timely making alerts of YFV activity, start emergency risk communication in risk communities and strengthening vaccination campaigns in target areas.
